# Homologous recombination is a force in the evolution of canine distemper virus

**DOI:** 10.1371/journal.pone.0175416

**Published:** 2017-04-10

**Authors:** Chaowen Yuan, Wenxin Liu, Yingbo Wang, Jinlong Hou, Liguo Zhang, Guoqing Wang

**Affiliations:** 1 College of life and health sciences, Northeastern University, Shenyang, Liaoning, China; 2 Laboratory of Hematology, Affiliated hospital of Guangdong Medical College, Zhanjiang, Guangzhou, China; 3 Center for Animal Disease Emergency of Liaoning province, Shenyang, Liaoning, China; Beijing Institute of Microbiology and Epidemiology, CHINA

## Abstract

Canine distemper virus (CDV) is the causative agent of canine distemper (CD) that is a highly contagious, lethal, multisystemic viral disease of receptive carnivores. The prevalence of CDV is a major concern in susceptible animals. Presently, it is unclear whether intragenic recombination can contribute to gene mutations and segment reassortment in the virus. In this study, 25 full-length CDV genome sequences were subjected to phylogenetic and recombinational analyses. The results of phylogenetic analysis, intragenic recombination, and nucleotide selection pressure indicated that mutation and recombination occurred in the six individual genes segment (H, F, P, N, L, M) of the CDV genome. The analysis also revealed pronounced genetic diversity in the CDV genome according to the geographically distinct lineages (genotypes), namely Asia-1, Asia-2, Asia-3, Europe, America-1, and America-2. The six recombination events were detected using SimPlot and RDP programs. The analysis of selection pressure demonstrated that a majority of the nucleotides in the CDV individual gene were under negative selection. Collectively, these data suggested that homologous recombination acts as a key force driving the genetic diversity and evolution of canine distemper virus.

## Introduction

Canine distemper (CD) is caused by the canine distemper virus (CDV) that belongs to the Morbillivirus genus of the Paramyxoviridae family. The length of the CDV genome is 15,690 nucleotides (nt), and it possesses a single-stranded negative RNA encoding six nonoverlapping transcriptional units producing eight proteins [1, 2]. The genomic RNA that is tightly encapsulated by the nucleocapsid (N) protein serves as a template for transcription and replication by the viral polymerase (L) protein and its cofactor phosphoprotein (P). The N, P, and L proteins together with the viral RNA constitute the ribonucleoprotein (RNP) [3], which directs the sequential synthesis of capped and polyadenylated mRNAs from six transcriptional units or the replication of full-length encapsulated antigenomes [4]. The viral envelope contains two integral membrane proteins, fusion (F) and hemagglutinin (H), as well as, a membrane-associated protein [matrix (M)] that altogether establish the contact with the RNP [5]. The H glycoprotein facilitates the binding of the virus to the cell membrane; the F protein accomplishes the fusion of the two membranes, which enables the entry of the viral RNP into the cytoplasm [6].

Canine distemper is a highly contagious and lethal disease that affects several many carnivorous species. This virus infects a wide variety of animal families, such as Mustelidae (ferrets, minks, skunks, weasels, and badgers), *Procy onidae* (raccoons), Ursidae (bears and pandas), *Viver ridae* (civets, genets, and linsangs), Hyaenidae (hyenas), and Felidae (lions and tigers). The genome of CDV encodes the genes for the following components: matrix (M = 1008 bp), fusion (F = 1989 bp), hemagglutinin (H = 1824 bp), nucleocapsid (N = 1572 bp), polymerase (L = 6555 bp), and phosphoprotein (P = 1524 bp).

Recombination could serve as a critical process for the evolution of RNA viruses [7]. To date, studies regarding the recombination in the CDV genome are not available. Thus, in the present study, twenty-five full-length CDV genome sequences were subjected to phylogeny, recombination, and selection pressure analyses. As a consequence, six such CDV phylogroups were identified among which the homologous recombination occurred among, and the individual genes mentioned above were under negative selection pressure. These data indicated that one recombination and reassortment might contribute to the molecular diversity of CDV.

## Materials and methods

### Sequence data and alignment

The complete sequences of the CDV genome and its six genes (N, P, L, M, H, and F) were retrieved from GenBank. All sequences were aligned using ClustalW [8] and BioEdit [9], followed by visual confirmation. The virus name, GenBank accession number, isolation time, and place are summarized in Table 1.

**Table 1:** CDV genome sequences used in this study.

GenBank accession no.	Strain	Host	Country	Years
AY466011	98–2654	raccoon	USA	1998
AY443350	00–2601	raccoon	USA	2000
JX681125	HLJ1-06	fox	China	2006
AY542312	98–2646	raccoon	USA	1998
AY445077	98–2645	raccoon	USA	1998
EU716337	164071	*Canis familiaris*	USA	2004
AB687721	CYN07-hV	*Macaca fascicularis*	Japan	2008
JN896987	VR-1587	NA	NA	NA
AY649446	01–2689	raccoon	USA	2001
AY386315	5804	NA	USA	NA
AY386316	5804P	NA	USA	NA
AB687720	CYN07-dV	*Macaca fascicularis*	Japan	2008
KC427278	Hebei	mink	China	2008
HM852904	MKY-KM08	*Macaca mulatta*	China	2008
HM063009	Shuskiy	mink	Kazakhstan	1989
HM046486	Phoca/Caspian/2007	seal	Kazakhstan:	2007
GU138403	recombinant Snyder Hill	ferret	USA	2010
AF164967	A75/17	NA	USA	1999
EU726268	CDV3	mink	China	NA
AB475097	M25CR	NA	Japan	NA
AB462810	007Lm-1vp	Vero cells	Japan	NA
AB474397	007Lm	NA	Japan	NA
AB476401	011C	Canis lupus familiaris	Japan	NA
AB476402	50Con	Canis lupus familiaris	Japan	NA
AB475099	55L	Canis lupus familiaris	Japan	NA
KC802221	PDV/Wadden_Sea.NLD/1988	*Phoca vitulina*	Netherlands	1988

### Phylogenetic analysis

The gene sequences mentioned above were trimmed by MEGA V5.0 [10]. Seven transversional models with a proportion of invariable sites and a substitution model comprised of gamma-shaped distribution of rates across these sites (CDV genome = GTR+G+I, H = GTR+G, N = GTR+G, P = TrN+G, F = TIM+G, M = TrN+G, L = GTR+G+I) s were assessed by ModelTest v3.7 [11], PAUP* v4b10 (Swofford, 2003), and MrBayes v3.1.1 that explored the distance (neighbor-joining) and characteristics [Bayesian, maximum likelihood (ML)-based phylogenetic methods] of the nucleotides sequences [12]. The results of the analysis were confirmed using Bayesian and ML approaches with the MEGA 5.0 software package [10]. The phylogenetic tree was tested by bootstrapping with 1000 replicates. The phocine distemper virus (PDV) (GenBank accession number KC802221) was indicated as an outgroup in the seven models.

### Recombination analysis

The recombination events were assessed using RDP4.0 [13] and confirmed by Similarity Plot, Boot Scan, and Find Sites (using maximization of x^2^) implementing the sub-programs of SimPlot v3.5.1 [14] or identified by BLAST in GenBank. After the CDV genomes, viral H, N, P, M, F, and L genes were aligned using ClustalW [8]. The Phi test in SplitsTree 4.11 [15] provided statistically significant evidence for recombination. Subsequently, the ML trees were constructed by MEGA 5.0, and bootstrap (1000 replicates) values were shown. Shimodaira–Hasegawa (SH) test affirmed the statistical difference between the phylogenetic trees estimated from different regions. The statistical significance was set at p < 0.05. All the analyses were conducted in triplicate [16].

### Selection pressure analysis

The non-neutral selection was calculated by the ratio of nonsynonymous (dN) to synonymous (dS) substitutions using ML phylogenetic reconstruction and the general reversible nucleotide substitution model available through the Datamonkey web server [17]. To detect non-neutral selection, single-likelihood ancestor counting (SLAC), fixed-effects likelihood (FEL), internal fixed-effects likelihood (IFEL), and random effects likelihood (REL) within the HyPhy software package [18] were implemented in the Datamonkey. The significance levels were set at p = 0.1, p = 0.1, p = 0.1, respectively, and Bayes factor = 50 was used to estimate the rates of dN and dS within each codon. The values dN/dS > 1, dN/dS = 1 and dN/dS < 1 were used to define the positive selection (adaptive molecular evolution), neutral mutations, and negative selection (purifying selection), respectively.

## Results

### Phylogenetic relationships of CDVs

**Fig 1:**
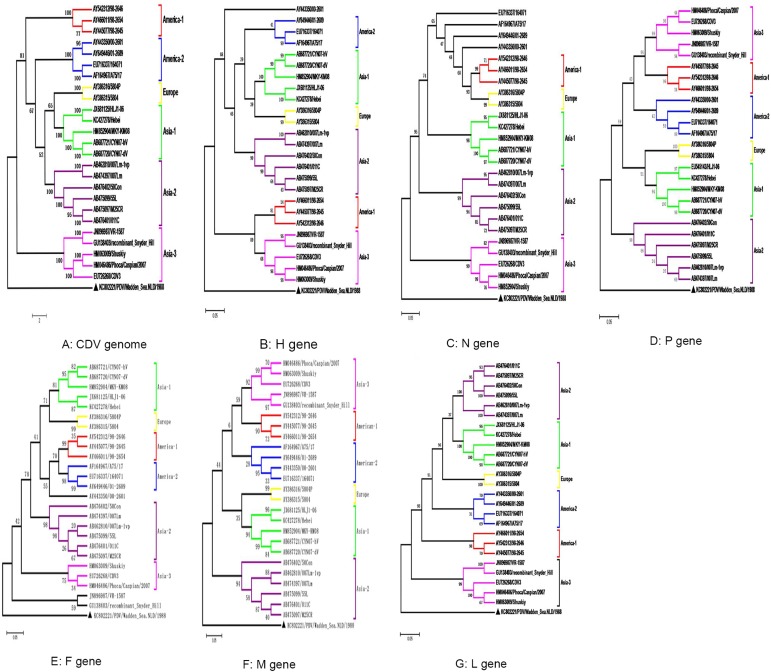
Phylogenetic tree analysis of 25 full-length CDV genomes. The distances were calculated by the ClustalW program with the MEGA 5.0 software package. The ML algorithm was used to generate the tree. Bootstrap values were calculated based on 1000 replicates. The result contains six groups: Asia-1, Asia-2, Asia-3, Europe, America-1, and America-2. Triangle (▲) indicates the out-group strains analyzed in this study.

All of the 25 full-length CDV strains originated from China, USA, or Japan, except 2 strains that were isolated from Kazakhstan. (Table 1). To determine the genetic relationships among the CDVs, seven phylogenetic trees were constructed (Fig 1) based on the available N, H, P, M, F, L segments and CDV genome sequences. The results showed that the CDVs could be divided into six lineages: Asia-1, Asia-2, Asia-3, Europe, America-1, and America-2. However, in the phylogenetic tree of the N gene, we can only find five lineages including America-1, Asia-1, Asia-2, and Asia-3. We also observed that one strain (AY443350/00-2601) in the H gene and two strains (GU138403/recombinant-Snyder-Hill, JN896987/VR-1587) in the F gene were independent.

### Recombination analysis of CDV using RDP4 and SimPlot programs

**Fig 2:**
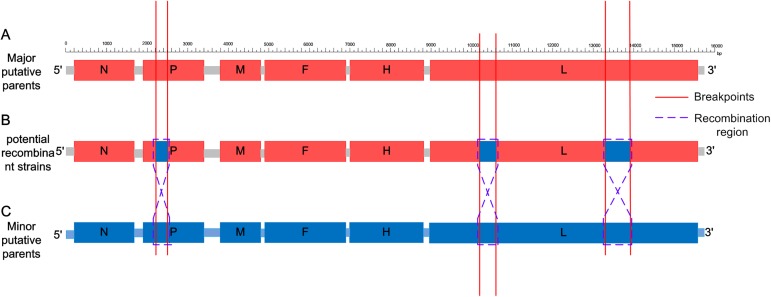
A schematic map of the genome for the recombinant strains of CDV. (A) Major putative parents for recombinant strains. (B) Potential recombinant strains of CDV. (C) Minor putative parents for recombinant strains. The same breakpoints in different genome schematics were labeled with a red line, and the recombinant fragments were labeled with a blue dotted line. The x-axis indicated the nucleotide position along the alignment (gap 100 bp).

In order to seek evidence for the natural recombination, the CDV complete genome sequence alignment dataset was analyzed by the RDP4 software package, which scanned the recombinant sequence. The six CDV isolates exhibited robust recombination signals. The p–values demonstrating the statistical significance of these programs were shown in S1 Table. The breakpoints were also confirmed by the RDP4 program [13]. Each of the recombination isolate encompassed two strains, which were suggested as the representatives of its putative parental lineages (Fig 2). To further validate the authenticity of the recombination events, SimPlot was employed that exhibited a result similar to that of the RDP4 program except for the breakpoint. The crossover sites depend on the size of the sliding window in SimPlot, and hence, the recombination breakpoints in RDP4 and SimPlot are different. The gold-standard bioinformatic approach for demonstrating the presence of recombination is a set of statistically incongruent phylogenetic trees [19]; thus, phylogenetic trees were also constructed using MEGA 5.0 to further determine the recombination events. Eventually, six groups of potential recombination events have been determined (Table 2).

**Table 2:** CDV strains with evidence for potential recombination.

Gene	Event	GenBank accession no.	Strain	Putative parent lineage(s)^*a*^	SimPlot identified breakpoint regions		RDP identified breakpoint regions	Final identified breakpoint regions	Country
Genome	I	JX681125	HLJ1-06	KC427278/HeBei; HM046486/Phoca/Caspian/2007	12771–14312		12922–14259	12922–14259	China
	II	AY466011	98–2654	EU726268/CDV3; EU716337/164071	2015–2833	7337–9131	2134–2504	2134–2504	USA
	III	AY542312	98–2646	EU726268/CDV3; EU716337/164071	2015–2833	7337–9131	2134–2504	2134–2504	USA
	IV	AY445077	98–2645	EU726268/CDV3; EU716337/164071	2015–2833	7337–9131	2134–2504	2134–2504	USA
	V	AB462810	007Lm-1vp	AY649446/01-2689; AB476401/011C	9846–10558		9789–10558	9846–10558	Japan
	VI	AB474397	007Lm	AY649446/01-2689; AB476401/011C	9846–10558		9789–10558	9846–10558	Japan
L gene	I	JX681125	HLJ1-06	KC427278/HeBei; HM046486/Phoca/Caspian/2007	3743–5297		3749–5255	3749–5255	China
	V	AB462810	007Lm-1vp	AY649446/01-2689; AB476401/011C	683–1562		620–1547	683–1547	Japan
	VI	AB474397	007Lm	AY649446/01-2689; AB476401/011C	683–1562		620–1547	683–1547	Japan
P gene	II	AY466011	98–2654	EU726268/CDV3; EU716337/164071	345–977		334–1075	345–977	USA
	III	AY542312	98–2646	EU726268/CDV3; EU716337/164071	345–975		333–1158	345–977	USA
	IV	AY445077	98–2645	EU726268/CDV3; EU716337/164071	345–977		334–1075	345–977	USA

^a^ The major and minor putative parental lineages of recombination isolates.

#### One intragenic recombinant was identified in CDV strain HLJ1-06 and its L gene

**Fig 3:**
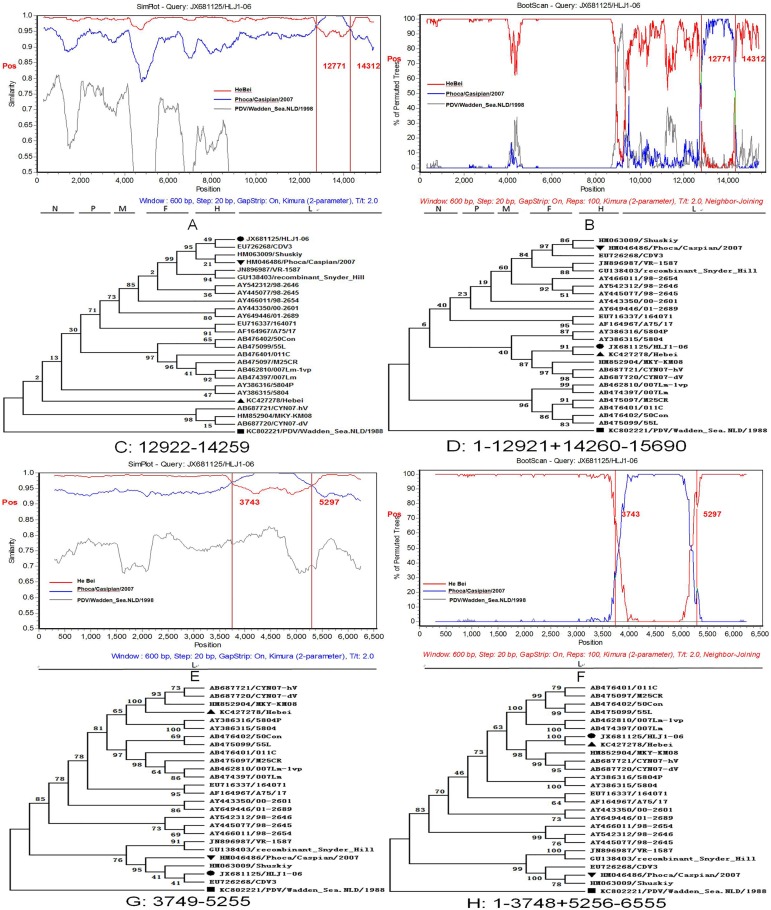
Analysis of recombination breakpoints in the CDV genome strain HLJ1-06 using the SimPlot program and MEGA 5.0 software package. (A) A similarity Plot analysis of the genome sequences of JX681125/HLJ1-06 and its putative parents (KC427278/HeBei and HM046486/ Phoca/Caspian/2007). The JX681125/HLJ1-06 was used as the query. The y-axis presented the percentage of identity with a window size of 600 bp and a step size of 20 bp. The vertical line indicated the breakpoint regions. (B) Boot Scan analysis of JX681125/HLJ1-06 and its parent sequences. The y-axis presented the percentage of permutated trees using a sliding window. (E) Similarity Plot analysis of the L gene sequences of JX681125/HLJ1-06 and its putative parents (KC427278/HeBei and HM046486/Phoca/Caspian/2007). (F) Boot Scan analysis of JX681125/HLJ1-06 and its parent sequences in L gene. The remaining are identical as in panels A and B. A PDV isolate, KC80222/PDV/Wadden_Sea.NLD/1998 was used as an outgroup. (C and D) The phylogenetic tree of the segment in different regions in the CDV genome from 12922–14259 and 1–12921 + 14260–15690 bp. (G and H) The phylogenetic tree of the segment in different regions in the L gene from 3749–5255 and 1–3748 + 5256–6555 bp. The recombinant strain and their major and minor putative parents are indicated with “●”, “▲”, and “▼”, respectively. The PDV isolates are marked with a “■”.

The analyses using RDP and SimPlot programs showed that the first potential recombinant event occurred in the strain (JX681125/HLJ1-06). The two strains (KC427278/Hebei and HM046486/ Phoca/Caspian/2007) isolated from China and Kazakhstan might be the major and minor putative parents, respectively, and the breakpoint regions of the potential recombinant strain, JX681125/HLJ1-06, were localized from 12771–14312 (in SimPlot) and 12922–14259 (in RDP4) bp. To further analyze the identified recombination event, the strain JX681125/HLJ1-06 was utilized as the query in the SimPlot program (Fig 3A). One potential breakpoint was localized at the parsimonious region, 12771–14312 bp, as identified by the maximization of X^2^. The sequence of HLJ1-06 showed greater affinity with one putative parent lineage of Hebei position 1–12770 and 14313–15690 bp than the other putative parent Phoca/Caspian/2007. However, the sequence of Phoca/Caspian/2007from 12771–14312 bp shared a greater similarity with HLJ1-06 than Hebei. An identical evidence was displayed by the Boot Scan result (Fig 3B). Owing to the difference in the breakpoint regions in SimPlot and RDP, we selected the total partial 12922–14259 bp region for analysis. The recombination events were further substantiated by constructing the phylogenetic tree using MEGA 5.0. In the putative non-recombinant regions (positions 1–12921 + 14260–15690), HLJ1-06 and Hebei were clustered into the same sub-lineage (Fig 3C), while Phoca/Caspian/2007 was grouped into a distinct sub-lineage. However, in the putative recombinant regions, 12922–14259 bp, the arrangement of the phylogenetic tree reflecting the relationship of the three isolates contradicted the previous tree (Fig 3D). The topology of the two phylogenetic trees around the breakpoint showed a significant statistical discrepancy when the PDV was included in the analyzed data (Shimodaira–Hasegawa test, p < 0.001) constituting a robust evidence for recombination.

In order to identify the gene and the portion of the segment that leads to the potential recombinant event, all the six individual genes of CDV were analyzed using RDP4, SimPlot, and MEGA 5.0 programs. The putative recombinant events occurring in the L gene were similar to that described in the CDV genome. The two potential recombinant events exhibited a high similarity among the recombinant strains, major and minor putative parents, except the breakpoints. The breakpoints in the L gene were located from 3743–5297 (in SimPlot) and 3749–5299 (in RDP4). We also analyzed this potential recombinant event with the method used for the CDV genome strain (JX681125/HLJ1-06) (Fig 3E–3H). Collectively, these analyses provided significant evidence for the recombination event described above.

#### Three similar intragenic recombinants were identified in the America-1 group of the CDV genome and P gene

The America-1 group contains three strains (AY445077/98-2645, AY542312/98-2646, and AY466011/98-2654). According to the analysis of the three genome sequences by RDP4 and SimPlot, three potential recombination events harbored identical breakpoint regions in the CDV genome (Table 2). The three sequences aligned using ClustalW and compared by the DNAStar program showed a high sequence similarity (> 99%) with each other (date not shown). Here, AY445077/98-2645 served as a representative strain for further analyses. Based on the results of RDP4 and SimPlot, the breakpoint regions of the potential recombinant strain (AY445077/98-2645) were located from 2015–2833 (SimPlot) and 2134–2504 (RDP4). The American and the Chinese strains, EU716337/164071 and EU726268/CDV3, respectively, act as the major and minor putative parents.

**Fig 4:**
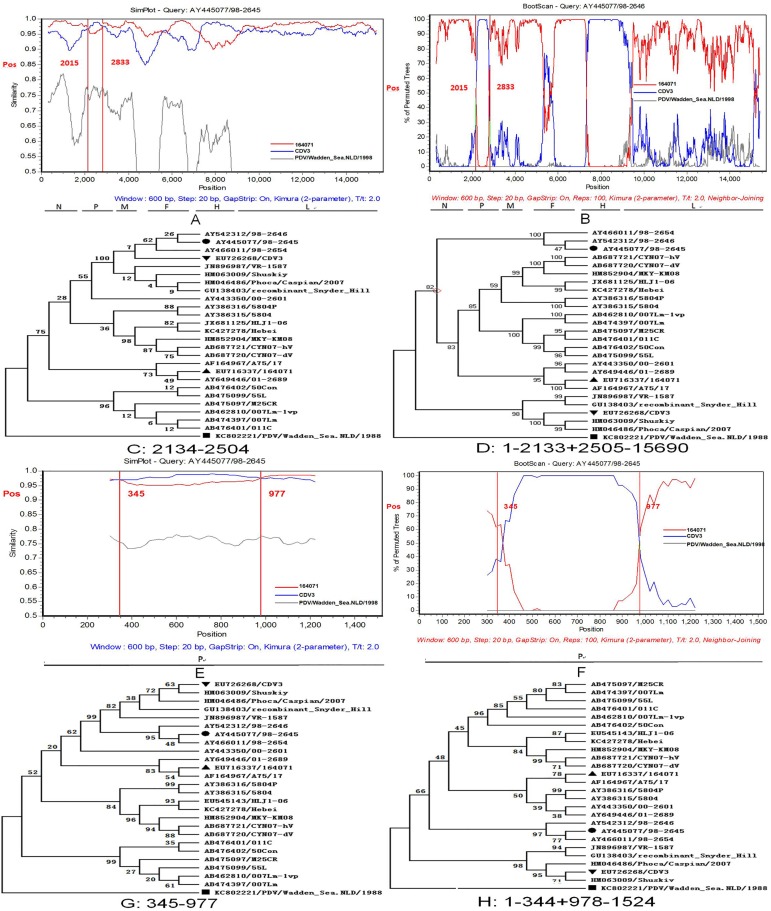
Evidence for homologous recombination in the CDV genome and L gene. (A) Similarity Plot analysis of the genome sequences of AY445077/98-2645 and its putative parents (EU716337/164071 and EU726268/CDV3). The AY445077/98-2645 was used as the query. The y-axis provided the percentage of identity with a window size of 600 bp and a step size of 20 bp. The vertical line indicated the breakpoint regions. (B) Boot Scan analysis of AY445077/98-2645 and its parent sequences. The y-axis presented the percentage of permutated trees using a sliding window. (E) The similarity plot analysis of the L gene sequences of AY445077/98-2645 and its putative parents (EU716337/164071 and EU726268/CDV3). (F) The Boot Scan analysis of AY445077/98-2645 and its parent sequences in the P gene. The remaining results are the similar as in panels A and B. A PDV isolate, KC80222/PDV/Wadden_Sea. NLD/1998 was used as an outgroup. (C and D) The phylogenetic tree of the segment in different regions in the CDV genome from positions 2134–2504 and positions 1–2133 + 2505–15690 bp. (G and H) The phylogenetic tree of the segment in different regions in the P gene from 345–977 and 1–344 + 978–1524 bp. The recombinant strain and their major and minor putative parents are indicated with “●”, “▲”, and “▼”, respectively. The PDV isolates are marked with a “■”.

To further analyze the identified recombination events, the AY445077/98-2645 strain was used as the query in the SimPlot program (Fig 4A). The result demonstrated that the sequence of AY445077/98-2645 showed a greater relatedness with one putative parent lineage of EU716337/164071 in the region from position 1–2014 and 2834–15690 bp than the other putative parent (EU726268/CDV3). However, the sequence, EU726268/CDV3, from 2015–2833 bp shared a greater similarity with AY445077/98-2645 than EU716337/164071. The identical evidence was provided by the Boot Scanning result (Fig 4B).

Subsequently, according to the potential recombinant events and cause of the differences in the breakpoint regions in SimPlot and RDP, we finally selected the conservative interval (2134–2504) for analysis. The phylogenetic trees were also constructed using MEGA 5.0 to determine the actual occurrences in the potential recombinant event. In the putative non-recombinant regions (positions 1–2133b + 2505–15690 bp), AY445077/98-2645 and EU716337/164071 were clustered into the same sub-lineage (Fig 4C), while EU726268/CDV3 was grouped into a distinct sub-lineage. However, in the putative recombinant regions, 2134–2504 bp, the arrangement of the phylogenetic tree reflecting the relationship of the three isolates was converse to the previous tree (Fig 4D).

In order to identify the gene and the position of the segment that leads to the potential recombinant events, all the six individual genes were analyzed in RDP4, SimPlot, and MEGA 5.0 programs. Interestingly, we found a potential recombinant event occurring in the P gene that was similar to that described in the potential recombinant event (AY445077/98-2645, breakpoint regions from 2134–2504 bp). Compared to the two potential recombinant events, these breakpoint regions exhibit a high similarity to the recombinant strain and the major and minor putative parents except the breakpoints. The breakpoint regions in the P gene were located from 345–977 (SimPlot) and 334–1075 (RDP4) bp. We also analyzed this potential recombination event with the method used in the CDV genome strain (AY445077/98-2645) and observed identical results (Fig 4E–4H).

#### Last two similar intragenic recombinants were identified in strains 007Lm-1vp and 007Lm of the CDV genome

In the case of the last two potential recombinant strains are AB462810/007Lm-1vp and AB474397/007Lm, the Japan strain AB476401/011C, and the American strain AY649446/01-2689 act as their major and minor putative parents. The two sequences of the recombinant strains were aligned using ClustalW and compared by DNAStar; we found > 99% homology (date not shown). Thus, we just select AB462810/007Lm-1vp as a representative strain for further analysis. Based on the results obtained by RDP4 and SimPlot, the breakpoint regions of the potential recombinant strain (AB462810/007Lm-1vp) were located from 9846–10558 (SimPlot) and 9789–10558 (RDP4).

**Fig 5:**
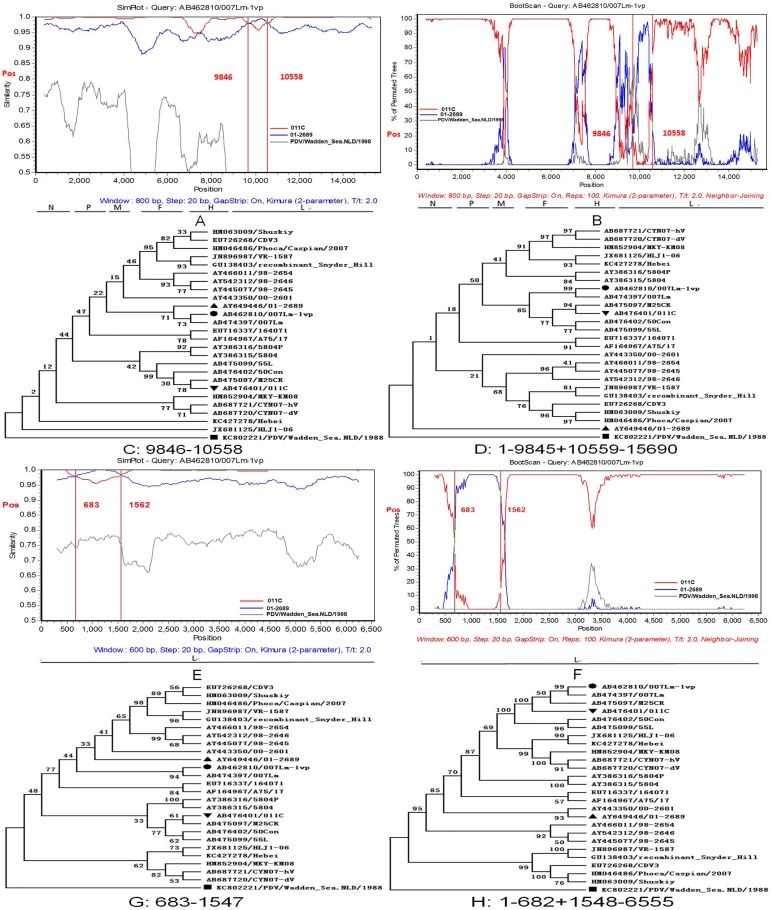
Analysis of recombination breakpoints in CDV genome strain 007Lm-1vp by SimPlot program and MEGA 5.0 software package. (A) Similarity Plot analysis of the genome sequences of AY462810/007Lm-1vp and its putative parents (AB476401/011C and AY649446/01-2689). The AY462810/007Lm-1vp was used as the query. The y-axis exhibited the percentage of identity with a window size of 600 bp (800 bp) and a step size of 20 bp. The vertical line indicated the breakpoint regions. (B) Boot Scan analysis of AY462810/007Lm-1vp and its parent sequences. The y-axis represented the percentage of permutated trees using a sliding window. (E) The similarity Plot analysis of the L gene sequences of AY462810/007Lm-1vp and its putative parents (AB476401/011C and AY649446/01-2689). (F) Boot Scan analysis of AY462810/007Lm-1vp and its parent sequences in the L gene. The remaining are similar as in panels A and B. A PDV isolate, KC80222/PDV/Wadden_Sea.NLD/1998 was used as an outgroup. (C and D) Phylogenetic tree of the segment in different regions in the CDV genome from 9846–10558 and 1–9845 + 10559–15690 bp. (G and H) Phylogenetic tree of the segment in different regions in the L gene from positions 683–1547 and positions 1–682 + 1548–6555 bp. The recombinant strain and their major and minor putative parents are indicated with “●”, “▲”, and “▼”, respectively; the PDV isolates are marked with a “■”.

To further analyze the identified recombination event, the AB462810/007Lm-1vp strain was used as the query in the SimPlot program (Fig 5A). This revealed that the sequence of AB462810/007Lm-1vp showed a greater affinity with one putative parent lineage of AB476401/011C than the other putative parent AY649446/01-2689 in the region 1–9719 + 10635–15690 bp. However, the sequence from 9720–10634 bp in AY649446/01-2689 shared a greater similarity with AB462810/007Lm-1vp than AB476401/011C. An identical evidence was provided by Boot Scanning (Fig 5B). Due to the difference of the breakpoint regions in SimPlot and RDP, we finally selected the partial 9846–10558 bp region for analysis. Then, the recombination events were further confirmed by constructing the phylogenetic tree using MEGA 5.0. In the putative non-recombinant regions (positions 1–9845 and 10559–15690 bp), AB462810/007Lm-1vp and AB476401/011C were clustered into the same sub-lineage (Fig 5C), while Phoca/Caspian/2007 was grouped into a distinct sub-lineage. However, in the putative recombinant regions 9846–10558 bp, the arrangement of the phylogenetic tree reflecting the relationship of the three isolates was in contrast to the previous tree (Fig 5D). The topology of the two phylogenetic trees around the breakpoint showed a significant statistical discrepancy when the PDV was included in the analyzed data (Shimodaira–Hasegawa test, p < 0.001).

In order to identify the gene and position of the segment leading to the potential recombinant events, all the six individual genes were analyzed in RDP4, SimPlot and MEGA 5.0 programs. Interestingly, we found a potential recombinant event occurring in the L gene which was the similar to that described in the potential recombinant event (AB462810/007Lm-1vp, breakpoint regions from 9846–10558 bp). Compared to the two potential recombinant events, these regions have a high similarity than that containing the recombinant strain, major and minor putative parents except the breakpoints. The breakpoint regions in the L gene were located from 683–1562 (in SimPlot) and 620–1547 (RDP4). We also analyzed this potential recombinant event with the method employed for the analysis of the CDV genome strain, AB462810/007Lm-1vp, and observed similar results (Fig 5E–5G).

### Selection pressure on the CDVs

The ratio of dN:dS substitutions/nucleotide site was estimated from an alignment of the six individual genes in CDV. The predominant selection pressure on the six individual genes was negative as reflected in a dN/dS value of < 1 (S2 Table). Using FEL (p = 0.1), 99% of the codons were under the negative selection pressure. However, we also found nine representative positive selection sites in the six individual genes of CDV; four positive selection sites were located in the H (positions 178), N gene (positions 456), M (positions 312), and L genes (positions 2137), respectively. Two positive selection sites (at positions 79 and 110) were detected in the F gene; whereas, three positive selection sites (at positions 148, 256 and 278) were detected in the P gene (FEL p-value < 1.0; SLAC p-value < 0.3) (S3 Table).

## Discussion

During the past two decades, despite the vaccination procedures adopted in the world, CDV continues to pose a severe threat to breeding foxes, raccoon dogs, minks, and domestic dogs [20, 21]. The prevalence of CDV may result in significant economic losses to the fur industry. Genetic diversity has been observed between wild-type CDVs and the vaccine strains [20, 22–26]. A majority of the live-modified CDV vaccine strains isolated could be dated to 1930–1950s [22, 27–29]. The diversity between the vaccine strains and wild-type CDVs may be attributed to several mechanisms, such as adapted to new host species [30, 31], antigenic escape [27, 32–34] and/or genetic recombination between wild-type strains [35], variedly driving the evolution of the virus. However, which intragenic recombination occurs and whether it contributes to the evolution of the virus is yet unclear.

In this study, 25 full-length CDV strains isolated from breeding foxes, raccoon, minks, seal, ferret, *Macaca fascicularis*, *Macaca mulatta*, and Canis lupus familiaris were sequenced and analyzed. Based on the phylogenetic analysis of the CDV genomes, all the CDV isolates were divided into six lineages: Asia-1, Asia-2, Asia-3, Europe, America-1, and America-2. According to the phylogenetic analysis, all the CDV isolates detected in naturally occurring cases clustered according to the geographical distribution. Comparing the phylogenetic trees between CDV genome sequences and six individual gene sequences, the topology of the phylogenetic trees in every individual gene were found to have a high identity with the phylogenetic tree of the genome sequence. Furthermore, the sequence homology of the CDV genome exhibited a high similarity than the six individual genes.

Homologous recombination drives genotype diversity in some RNA viruses [36–38]. Recombination, especially between different serotypes, may accelerate the evolution of the virus effectuating a rapid change in its epidemiology [39]. In this study, nine groups of potential recombination events were detected by RDP4 and SimPlot programs, and the six potential recombination events included six recombination strains (JX681125/HLJ1-06, AY445077/98-2645, AY542312/98-2646, AY466011/98-2654, AB462810/007Lm-1vp, and AB474397/007Lm). According to the phylogenetic analysis, the JX681125/HLJ1-06 strain belongs to the group of Asia-1. The other three strains, AY445077/98-2645, AY542312/98-2646, and AY466011/98-2654, belonged to the group of America-1. The remaining two strains, AB462810/007Lm-1vp and AB474397/007Lm, belonged to the group of Asia-2. Similar to that reported previously [40], we confirmed that JX681125/HLJ1-06, AB462810/007Lm-1vp, and AB474397/007Lm were wild-type strains. On the contrary, the other three strains, AY445077/98-2645, AY542312/98-2646, and AY466011/98-2654 were vaccine strains but not artificial [41]. Moreover, it could be speculated that the strains of the same group may have a high homology, thereby leading to similar recombination events. Thus, the six sequences were aligned using ClustalW and compared by DNAStar. Consequently, we found that the three strains, AY445077/98-2645, AY542312/98-2646, and AY466011/98-2654, showed a high sequence similarity (> 99%) with each other (date not shown), as well as, the other two strains (AB462810/007Lm-1vp and AB474397/007Lm). Finally, the three strains, X681125/HLJ1-06, AY445077/98-2645, and AB462810/007Lm-1vp, were selected as representatives for further analyses.

The RDP4 and SimPlot analyses detected the three recombination regions. The fist recombination region was located from 12922–14259 bp in the JX681125/HLJ1-06 strain and the two strains, KC427278/Hebei and HM046486/ Phoca/Caspian/2007, might be the major and minor putative parents. The second recombination region was localized in the position 2135–2504 bp in the AY445077/98-2645 strain and the two strains, EU716337/164071 and EU726268/CDV3, might be the major and minor putative parents. The last recombination region was found at 9846–10558 bp in the AB462810/007Lm-1vp strain and two strains, AB476401/011C and AY649446/01-2689, might act as the major and minor putative parents.

In order to explore which gene can lead to the intragenic recombination, all the strains of six individual genes were also analyzed by RDP4 and SimPlot programs. Three parental recombination events were detected in P and L gene, they were the same as what we found in the strains (JX681125/HLJ1-06, AY445077/98-2645, and AB462810/007Lm-1vp) and no parental recombination event was found in other genes (H, N, F, and M gene). Previously report said the L gene exhibits a highly conserved property [40]. However, we found that P and L genes are the major recombination regions and we speculate that the L gene of Canine distemper virus may not have a high conservativeness.

In order to further illustrate the possibility of the six potential recombination events, a set of statistically incongruent phylogenetic trees (two for every event) were analyzed for their presence. [19]. The first phylogenetic tree was constructed in the recombination region and the second in the non-recombination region. The results indicated that the recombinant interval had a specific group of parents (major and minor putative parents), and the non-recombination interval had another group of parents, which might statistically support the phylogenetic recombination signal. These results suggested that all the phylogenetic trees supported the recombination result obtained from RDP4 and SimPlot analyses. In 2003, Holmes et al. indicated that the rates of homologous recombination in negative-sense RNA viruses were extremely low; however, the possibility of recombination in CDV should be investigated further [42]. The current study confirms the existence of homologous recombination in CDVs.

The data regarding the host of the virus are essential to understand the genetic diversity and evolution of CDV. In this study, we found that the hosts of recombination strains and their major and minor putative parents were different. The host of the first recombination strain JX681125/HLJ1-06 was a fox, and the hosts of its major (KC427278/Hebei) and minor putative (HM046486/ Phoca/Caspian/2007) parents were mink and seal, respectively. The host of the second recombination strain AY445077/98-2645 was a raccoon, but the hosts of its major (EU716337/164071) and minor putative (EU726268/CDV3) parents were mink and *Canis familiaris*, respectively. The host of the third recombination strain AB462810/007Lm-1vp was Vero cell, and the hosts of its major (AB476401/011C) and minor putative (AY649446/01-2689) parents were raccoon and Canis lupus familiaris. Therefore, we may speculate that CDV isolated from different species may occur in intragenic recombination and contribute towards the evolutionary process in the virus.

RNA viruses are genetically flexible. During the replication of their genomes, the nucleotide substitutions occur at high frequencies presumably to allow rapid adaptations to various selection pressures [43]. In the current study, considering that the six individual genes were vital to CDV, we assessed the nucleotide selection pressure in the six individual genes (H, F, P, N, M, L). No previous studies suggested whether these six individual genes were under negative or positive selection. In this study, the mean ratio of dN and dS rates (dN/dS) for the six individual genes were both < 1, and the rate for the six individual genes was M (0.074) < L (0.082) < N (0.095) < H (0.263) < F (0.310) < P (0.352) (S2 Table). These result indicated that the six individual genes were under purifying selection; P was under stronger negative selection than other genes, while M exhibited a highly conserved property. This finding strongly correlated with the higher evolutionary rate of P compared to other genes and postulated the correlation of the selection pressure and the rate of evolution [44, 45]. We also found nine positive selection sites (H-178, N-456, M-312, L-2137, F-79/110, and P-148/256/278), especially, positive natural selection involved in immune escape could cause similar sequences to evolve in unrelated strains.

In conclusion, six phylogroups and homologous recombination events, occurring in the CDVs, were identified based on the CDV genome and their gene segments. The majority of CDVs underwent a negative selection pressure. This information provides a valuable reference for the study of molecular epidemiology of CDVs.

## Supporting information

S1 Table CDV strains with evidence for potential recombination in RDP analysis. (DOCX)

S2 Table The positive-selection sites represented by codons in six individual genes of CDV. (DOCX)

S3 Table Evidence for positive and negative selection using four detection methods. (DOCX)
