# Supplementary material for: Homologous recombination is a force in the evolution of canine distemper virus
Source: PLoS One. 2017 Apr 10;12(4):e0175416. doi: 10.1371/journal.pone.0175416 (PMC5386261; doi:10.1371/journal.pone.0175416)
Supplement: S1 Table — (DOCX) [file pone.0175416.s001.docx]

**S1 Table. CDV strains with evidence for potential recombination in RDP analysis**

| **CDV** | **Event** | **RDP** | **GENECONV** | **BOOTSCAN** | **MaxChi** | **CHIMAERA** | **SISCAN** | **PhylPro** | **LARD** | **3seq** |
| --- | --- | --- | --- | --- | --- | --- | --- | --- | --- | --- |
| Genome | Ⅰ | 9.461 E-70 | 8.878 E-70 | 4.927 E-72 | 3.066 E-18 | 1.783 E-18 | 1.027 E-18 | NA | NA | 5.280 E-51 |
|  | Ⅱ | 1.058 E-07 | 1.012 E-06 | 2.221 E-08 | 1.233 E-02 | 2.635 E-02 | 4.606 E-05 | NA | NA | NA |
|  | Ⅲ | 1.058 E-07 | 1.012 E-06 | 2.221 E-08 | 1.233 E-02 | 2.635 E-02 | 4.606 E-05 | NA | NA | NA |
|  | Ⅳ | 1.058 E-07 | 1.012 E-06 | 2.221 E-08 | 1.233 E-02 | 2.635 E-02 | 4.606 E-05 | NA | NA | NA |
|  | Ⅴ | 2.874 E-29 | 6.444 E-28 | 2.701 E-29 | 2.095 E-08 | 2.052 E-08 | 1.115 E-08 | NA | NA | 5.744 E-06 |
|  | Ⅵ | 2.874 E-29 | 6.444 E-28 | 2.701 E-29 | 2.095 E-08 | 2.052 E-08 | 1.115 E-08 | NA | NA | 5.744 E-06 |
| L gene | Ⅰ | 5.242 E-43 | 1.941 E-42 | 2.541 E-45 | 1.085 E-18 | 6.379 E-19 | 1.791 E-19 | NA | NA | 6.894 E-46 |
|  | Ⅴ | 8.594 E-51 | 4.444 E-47 | 3.735 E-50 | 9.102 E-21 | 3.436 E-07 | 4.704 E-27 | NA | NA | 7.164 E-36 |
|  | Ⅵ | 8.594 E-51 | 4.444 E-47 | 3.735 E-50 | 9.102 E-21 | 3.436 E-07 | 4.704 E-27 | NA | NA | 7.164 E-36 |
| P gene | Ⅱ | 1.320 E-02 | 1.136 E-02 | 1.180 E-03 | 3.850 E-06 | 4.892 E-06 | 6.800 E-06 | NA | NA | 1.363 E-05 |
|  | Ⅲ | 1.320 E-02 | 1.136 E-02 | 1.180 E-03 | 3.850 E-06 | 4.892 E-06 | 6.800 E-06 | NA | NA | 1.363 E-05 |
|  | Ⅳ | 1.320 E-02 | 1.136 E-02 | 1.180 E-03 | 3.850 E-06 | 4.892 E-06 | 6.800 E-06 | NA | NA | 1.363 E-05 |
