# Supplementary material for: Homologous recombination is a force in the evolution of canine distemper virus
Source: PLoS One. 2017 Apr 10;12(4):e0175416. doi: 10.1371/journal.pone.0175416 (PMC5386261; doi:10.1371/journal.pone.0175416)
Supplement: S2 Table — (DOCX) [file pone.0175416.s002.docx]

**S2 Table. Evidence for positive and negative selection using four detection methods**

| CDV genome | Selection  pressure analysis | SLAC | FEL | REL | IFEL |
| --- | --- | --- | --- | --- | --- |
|  | Positive selection | 0 | 2 | 0 | 3 |
| H gene | Negative selection | 27 | 83 | 18 | 57 |
|  | Overall dN/dS | 0.263 | NS | 0.584 | NS |
|  | Positive selection | 0 | 0 | 5 | 1 |
| N gene | Negative selection | 17 | 59 | 0 | 41 |
|  | Overall dN/dS | 0.095 | NS | 0.144 | NS |
|  | Positive selection | 0 | 5 | 0 | 7 |
| F gene | Negative selection | 15 | 73 | 9 | 46 |
|  | Overall dN/dS | 0.310 | NS | 0.659 | NS |
|  | Positive selection | 0 | 3 | 7 | 3 |
| P gene | Negative selection | 10 | 41 | 0 | 24 |
|  | Overall dN/dS | 0.352 | NS | 230,556,000 | NS |
|  | Positive selection | 0 | 0 | 1 | 0 |
| M gene | Negative selection | 13 | 38 | 97 | 23 |
|  | Overall dN/dS | 0.074 | NS | 2,577,230 | NS |
|  | Positive selection | 0 | 1 | 16 | 2 |
| L gene | Negative selection | 117 | 300 | 65 | 218 |
|  | Overall dN/dS | 0.082 | NS | 0.217 | NS |
