# Supplementary material for: Homologous recombination is a force in the evolution of canine distemper virus
Source: PLoS One. 2017 Apr 10;12(4):e0175416. doi: 10.1371/journal.pone.0175416 (PMC5386261; doi:10.1371/journal.pone.0175416)
Supplement: S3 Table — (DOCX) [file pone.0175416.s003.docx]

**S3 Table. The positive-selection sites represented by codons in** **six individual genes of CDV**

|  | SLAC | FEL | REL | IFEL | Common |
| --- | --- | --- | --- | --- | --- |
| H gene | NA | 178/549 | NA | 178/542/585 | 178 |
| N gene | NA | 11/27/456/511/512 | NA | 456 | 456 |
| F gene | NA | 27/43/51/79/110 | NA | 19/51/58/79/99/110/513 | 79/110 |
| P gene | NA | 148/256/287 | 83/148/195/221/256/278/287 | 148/256/287 | 148/256/278 |
| M gene | NA | NA | NA | 312 | 312 |
| L gene | NA | 2137 | 27/37/252/603/611/618/1323/1385/1707/1708/1711/1943/2076/2101/2137/2157 | 2076/2137 | 2137 |
